# A Case of Hour-Glass Contraction after the Third Stage of Labor

**Published:** 1886-10

**Authors:** R. W. Skipper

**Affiliations:** Bend, Texas


### A CASE OF HOUR-GLASS CONTRACTION AFTER THE THIRD STAGE OF LABOR.

*By R. W. Skipper, M. D., Bend, Texas.*

[For Daniel's Texas Medical Journal.]

I WAS called to wait on Mrs. G., in labor with her seventh child; arrived at 3 a. m.; found her having regular pains, and all going on well. At 5 a. m. she was delivered of a fine female child. In twenty minutes the secundines were brought away by the combined methods of traction and expression, so well known to all the readers of this article. Looking well to the condition of the uterus, and attending to other duties incident upon such occasions, I took my seat, to remain the length of time thought necessary. In about three-quarters of an hour the patient said she was flooding, and, on looseing the bandage, I found the uterus large and flabby. Grasping it with the cold wet hands, I compelled it to contract; and gave a full dose of fluid extract ergot, to insure against further trouble of like character. I reapplied the compress and binder, and again left her bed. She said the womb was not contracted, after a short interval, and again I found her flooding, but not severely. This time I made a vaginal examination, to ascertain if there were any shreds of membrane, or other cause for the hemorrhage than uterine inertia, and found none. From the time the organ began to contract this time, the lady suffered with the most terrific after-pains. The womb contracted in that peculiar way, known as "hour-glass" contraction; the external os being dilated, while the internal was fast closed, and the organ small, and much elongated, extended above the umbilicus. The patient's suffering was so great that I gave her a dose of morphia, and soon the pains subsided, leaving the organ firmly contracted, however, in the peculiar form above mentioned. I did not interfere, but after remaining until 10 o'clock a. m., five hours from birth of child, I gave orders to the husband to send for me at once if anything went wrong. I saw the patient at 6 p. m. She told me that she had just had some almost painless contractions, and there had heen expelled from the uterus some firm coagula, and that the organ then resumed its natural form and size, and she had no more trouble. Her getting up has been a good one, there being nothing abnormal in the case, except a slight swelling, with tenderness along the leg, where there had been some varicose veins. The patient had a mild attack of intermittent fever three weeks before confinement, which had been promptly arrested. She had had a severe attack of scorbutus twenty months before.

